# Incidence of non-typhoidal *Salmonella* invasive disease: A systematic review and meta-analysis

**DOI:** 10.1016/j.jinf.2021.06.029

**Published:** 2021-07-11

**Authors:** Christian S. Marchello, Fabio Fiorino, Elena Pettini, John A. Crump, Vacc-iNTS Consortium Collaborators

**Affiliations:** a Centre for International Health, University of Otago, PO Box 56, Dunedin 9054, New Zealand; b University of Siena, Siena, Italy

**Keywords:** Systematic review, Meta-analysis, Non-typhoidal *Salmonella*, Incidence

## Abstract

**Objectives:**

We sought to collate and summarize high-quality data on non-typhoidal *Salmonella* invasive disease (iNTS) incidence to provide contemporary incidence estimates by location and year.

**Methods:**

We systematically searched the databases Embase + MEDLINE, Web of Science, and PubMed for articles published on the incidence of iNTS from inception of the database through 8 May 2020 with no language, country, date, or demographic restrictions applied. A meta-analysis was performed to report pooled iNTS incidence as a rate of cases per 100,000 per year.

**Results:**

Among 13 studies eligible for analysis, there were 68 estimates of incidence. Overall pooled incidence (95% CI) was 44.8 (31.5–60.5) per 100,000 persons per year. When stratified by region, pooled incidence was significantly higher in Africa than Asia, 51.0 (36.3–68.0) compared to 1.0 (0.2–2.5), respectively. Incidence was consistently higher in children aged <5 years compared with older age groups. Incidence displayed considerable heterogeneity in both place and time, varying substantially between locations and over consecutive years in the same location.

**Conclusions:**

iNTS incidence varies by region, location, age group, and over time. Concerted efforts are needed to address the limited high-quality data available on iNTS disease incidence.

## Introduction

Non-typhoidal *Salmonella* (NTS) are an important cause of self-limited diarrheal disease often transmitted by food or water.^1^ However, in some patients NTS cause serious, life-threatening invasive infections involving the bloodstream, meninges, and other normally sterile sites.^2,3^ Patients with non-typhoidal *Salmonella* invasive disease (iNTS) often present with a non-specific febrile illnesses in the absence of recent or current diarrhea that is difficult to distinguish from other infectious diseases including malaria and typhoid fever.^2^ iNTS is a serious illness with a case fatality ratio of approximately 15%^40^ and was estimated to have caused 535,000 illnesses and more than 77,000 deaths in 2017.^4^ In a recent systematic review on the prevalence of community-onset bloodstream infections (BSI), NTS were among the most frequently isolated pathogens.^5^ *Salmonella enterica* subspecies *enterica* serovars Typhimurium and Enteritidis, accounted for more than 80% of serovars causing iNTS.^5,6^

Regionally, iNTS disease is concentrated in sub-Saharan Africa where it is a major cause of illness and death.^4^ Host risk factors including HIV, malaria, and malnutrition are thought to drive the disproportionate burden of iNTS in Africa compared to other regions.^7,8^ Treatment is proving increasingly problematic with widespread antimicrobial resistance among NTS isolates.^9^ *Salmonella* Typhimurium sequence type (ST) 313 accounts for the majority of *Salmonella* Typhimurium causing invasive disease in Africa,^10^ is predominately multi-drug resistant, and may also be extensively-drug resistant.^11^ Additionally, vaccine development has been slow to progress because of the limited data on burden of disease, as well as economic and technical challenges.^12^

In 2010 and 2017 population-based surveillance or national surveillance data were reviewed, and extrapolated to areas without incidence data based on host risk factors.^4,13,14^ However, a number of studies have been published since that time. We sought to collate and summarize high-quality data on iNTS incidence to provide contemporary incidence estimates by location and year for policymakers to support investments in vaccine development and non-vaccine intervention efforts.

## Methods

### Search strategy

We performed a search of the databases Embase + MEDLINE, Web of Science, and PubMed for articles published on the incidence of iNTS from inception of the database through 8 May 2020. No language, country, date, or demographic restrictions were applied to the search strategy (Box 1 and Supplementary Appendix A). We used key words of non-typhoidal *Salmonella*, non-Typhi, salmonellosis, incidence, epidemiology, burden, and specific serovars including Typhimurium, Enteritidis, Heidelberg, Dublin, Choleraesuis, Newport, Virchow, Concord, Brancaster, Freetown, Infantis, and Isangi. Specific serovars were selected based on previous reviews of prevalence of bloodstream infections^5,6^ and knowledge (JAC and CSM) of common *Salmonella* serovars that cause iNTS. Additionally, we screened citations of included full text articles. The Preferred Reporting Items for Systematic Reviews and Meta-Analyses was followed.^15^ The protocol was submitted to PROPSERO International Prospective Register of Systematic Reviews on 14 May 2020 and registered on 10 July 2020 (CRD42020186362). As an analysis of published data, this study was exempt from requiring institutional review board approval.

### Study selection

We included study designs based on active household or population-based surveillance, sentinel site surveillance using healthcare utilization surveys to adjust for under-ascertainment (i.e., hybrid surveillance or multiplier studies),^16,17^ prospective observational studies, or vaccine clinical trials for other invasive bacterial diseases with relevant control arm data. Studies recruited participants of any age reporting the number of cases of iNTS identified using cultures of a normally sterile site (e.g., blood, bone marrow) for confirmation. Raw data were required to calculate incidence rate as number of cases per 100,000 per year. We excluded study designs based on case reports, case series, and surveillance studies where collection of blood cultures from febrile patients was not systematic. We also excluded studies using only clinical indication (i.e., symptoms and signs), culture of a non-sterile site (e.g., stool or urine), or serology alone to classify a case of iNTS.

Text files for each database search result were downloaded and imported into Endnote X8 (Clarivate Analytics, Boston, MA, United States) and combined into one reference list. Duplicates of titles and abstracts were removed by Endnote, and uploaded to the online systematic review tool Rayyan (Qatar Computing Research Institute, Doha, Qatar) for screening.^18^ Titles and abstracts, and full text were screened in parallel for inclusion (CSM, FF, and EP). Data were then abstracted (CSM, FF, and EP) using Google Forms (Google LLC, Mountain View, CA, USA). The data abstraction form is available in Supplementary Appendix B. A third author (JAC) was consulted when discrepancies could not be resolved through discussion and reviewed the final dataset for completeness and accuracy.

### Data abstraction and analysis

Abstracted study characteristics included study country and location, United Nations (UN) region and sub-region, study design, data collection start and end date, duration of surveillance in months, normally sterile sites cultured, eligibility criteria for culture request, and age group of participants (children ≤ 15 years, adults >15 years, or mixed ages). Age groups were categorized based on inclusion criteria or age range data provided in results.

Study designs were stratified into two groups: (1) Active, household, or population-based surveillance or hybrid surveillance that used multipliers for adjustment, and (2) Unadjusted sentinel site surveillance. Hybrid surveillance studies were defined as those using one or more of the three multipliers described by Andrews and colleagues to adjust the crude incidence for under-ascertainment.^16^ These multipliers made adjustments for culture sensitivity, enrollment capture, or facility coverage estimated by an household survey. We defined unadjusted sentinel site surveillance as all other studies that were not active, population-based, or hybrid surveillance. To be eligible, unadjusted surveillance studies were required to have well-defined catchment population information where we were confident that the authors were able to capture a large proportion of potential iNTS cases through systematic testing.

Guided by bias assessment tools for prevalence, incidence, and non-randomized studies,^19–21^ we assessed the risk of bias in two main domains. For the selection and recruitment domain, we evaluated study design, incidence multipliers, and patient selection. In the measurement and reporting domain, we evaluated eligibility criteria for receiving a culture, data for incidence calculations, and microbiology methods. Each domain question was scored low or high risk for bias, and an overall score of low, moderate, or high risk of bias was assigned to each study. Definitions for scoring and each question are provided in Supplementary Appendix C.

We recorded the number of cases of iNTS, including by serovar when available, the population under surveillance, and the duration of surveillance in months. The number of cases were divided by the number of months of surveillance and then multiplied by 12 to calculate the number of cases per year. Cases per year were then divided by the population under surveillance and multiplied by 100,000 to report incidence as a rate of cases per 100,000 per year. All incidence data presented were per 100,000 persons per year, unless otherwise noted. When data were available for multiple study years, incidence was calculated by individual year when it was possible to do so. We performed a meta-analysis in MetaXL version 5.3 (Epigear International) using DerSimonian-Lairdrandom-effects model with double arcsine transformation to report pooled incidence estimates.^22^ We evaluated heterogeneity using Cochran Q-test and I^2^.

## Results

**Fig. 1.**
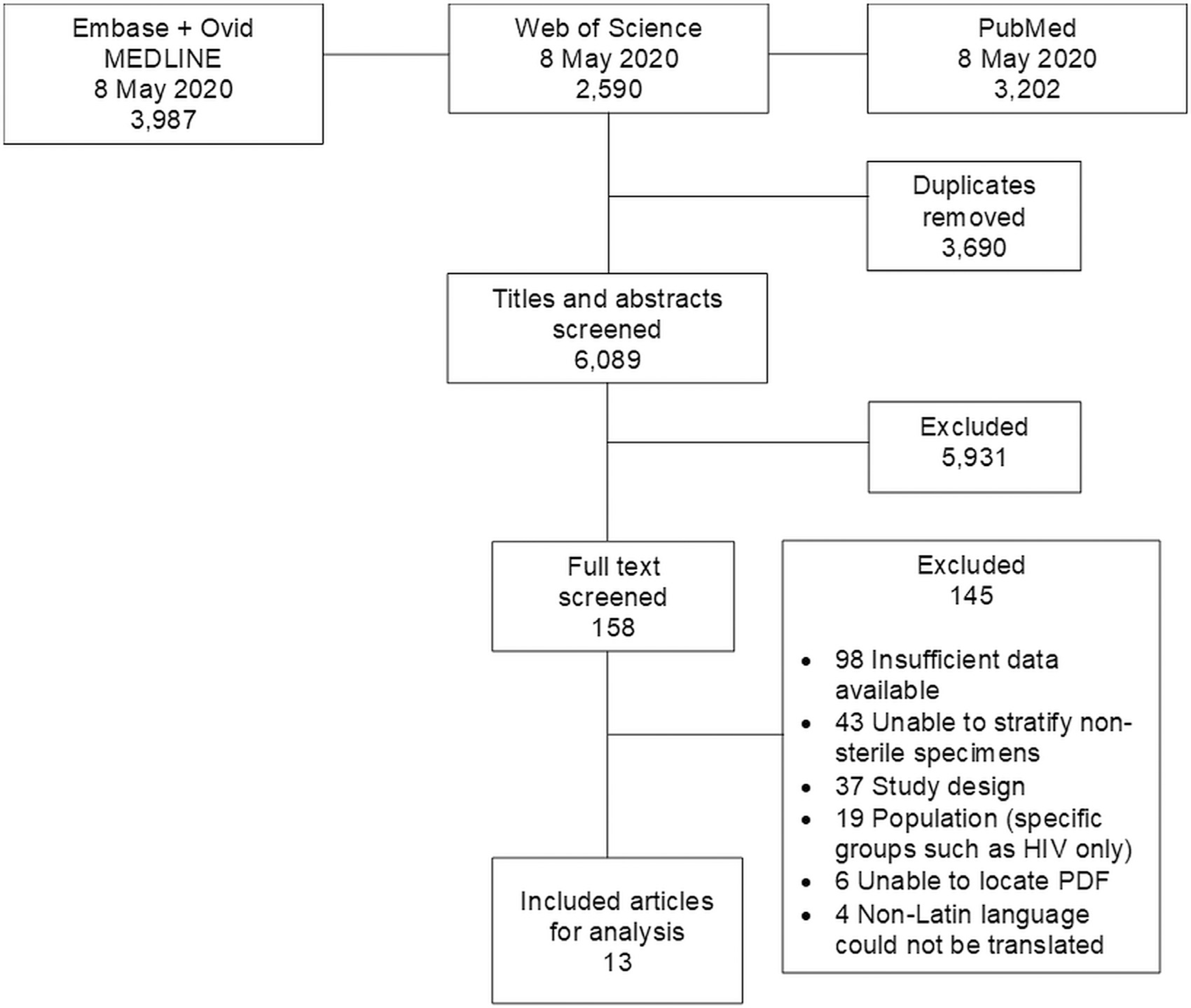
Preferred reporting items for systematic reviews and meta-analyses flow diagram of search strategy and selection of articles for incidence of non-typhoidal *Salmonella* invasive disease, 1996–2016.

Our search strategy returned 9,779 articles (Fig. 1) to be screened. After 3,690 duplicate articles were removed, we screened 6,089 titles and abstracts for inclusion. Of these, 158 (2.6%) proceeded for full text review. We excluded 145 articles after reviewing the full text; the most common reason for exclusion was insufficient data available to calculate incidence. Thirteen articles were eligible for analysis.^23–35^

### Study characteristics and quality assessment

Among the 13 included studies, data were collected from 1 January 1996 through 31 December 2016 in 19 countries from Africa and Asia. There were no included studies from any other UN region (Table 1). Eight studies collected data either from multiple locations or during multiple consecutive years,^23–25,27,29,33–35^ resulting in 68 separate estimates of iNTS incidence. The median (range) population under surveillance was 571,000 (5,333 to 850,000). There were 63 (92.6%) incidence estimates from Africa and 5 (7.4%) from Asia. Of the 68 estimates, 53 (77.9%) were from the Eastern Africa sub-region. Among 53 estimates from Eastern Africa, 25 (47.2%) were from Kenya, 17 (32.1%) from Malawi, 6 (11.3%) from Tanzania, 2 (3.8%) from Madagascar, and one (1.9%) each from Ethiopia, Mozambique, and Uganda.

**Fig. 2.**
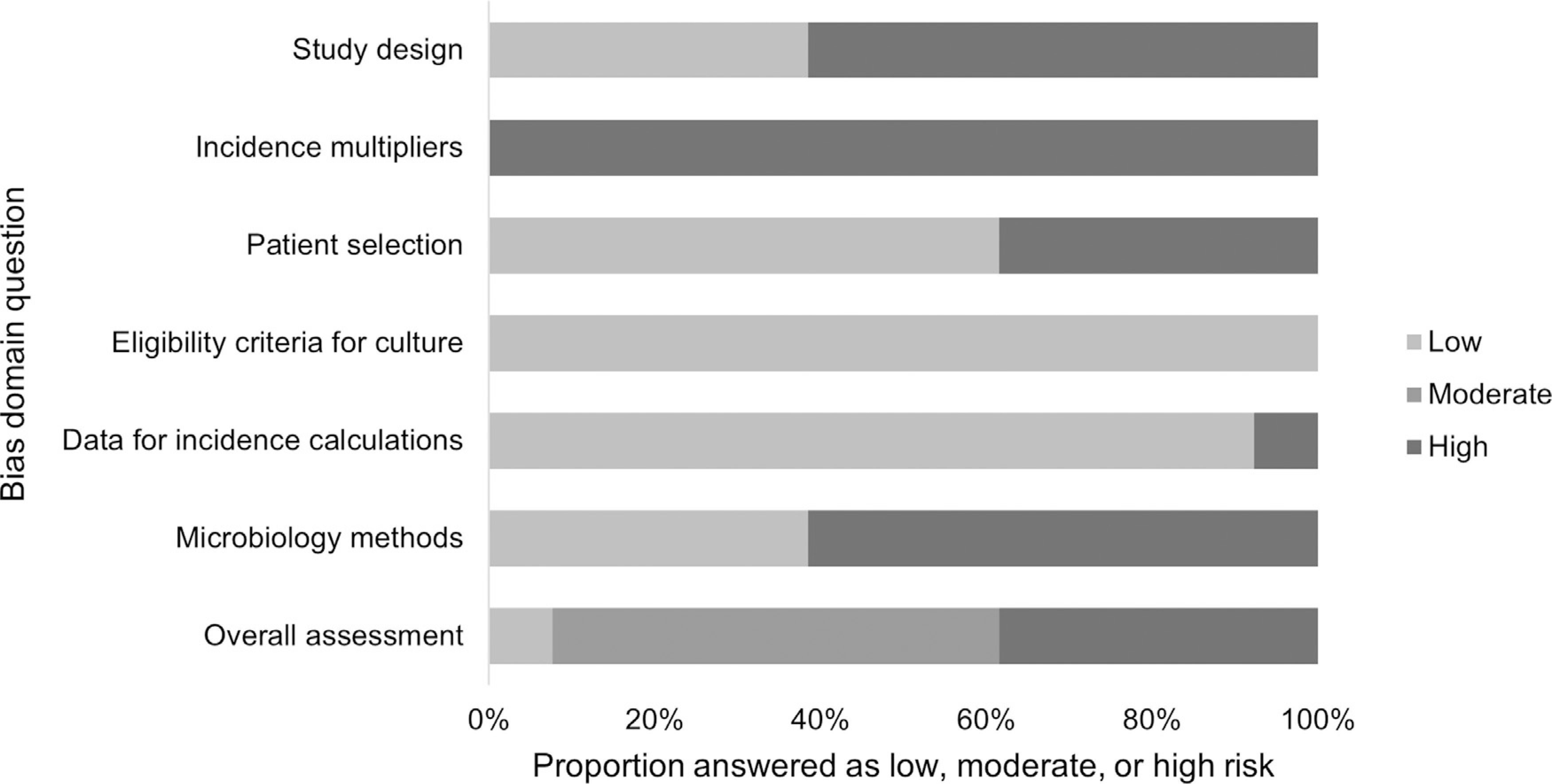
Quality assessment for risk of bias of included studies on incidence of non-typhoidal *Salmonella* invasive disease by domain, 1996 through 2016.

Data for 38 (55.9%) of 68 estimates were collected using an unadjusted sentinel site surveillance study design, while 30 (44.1%) estimates used hybrid surveillance design. There were no active, population-based studies that did not also include multipliers. Six studies reported a multiplier-adjusted incidence estimate.^23,27,30,31,34,35^ No article used all three multiplier adjustments described by Andrews, et al.^16^ In our bias assessment, five (38.5%) of 13 studies scored as high risk of bias,^23,25,30,31,33^ seven (53.8%) as moderate risk,^24,26–29,32,34^ and one (7.7%) as low risk (Fig. 2).^35^

### *Incidence of non-typhoidal* Salmonella *invasive disease*

**Fig. 3.**
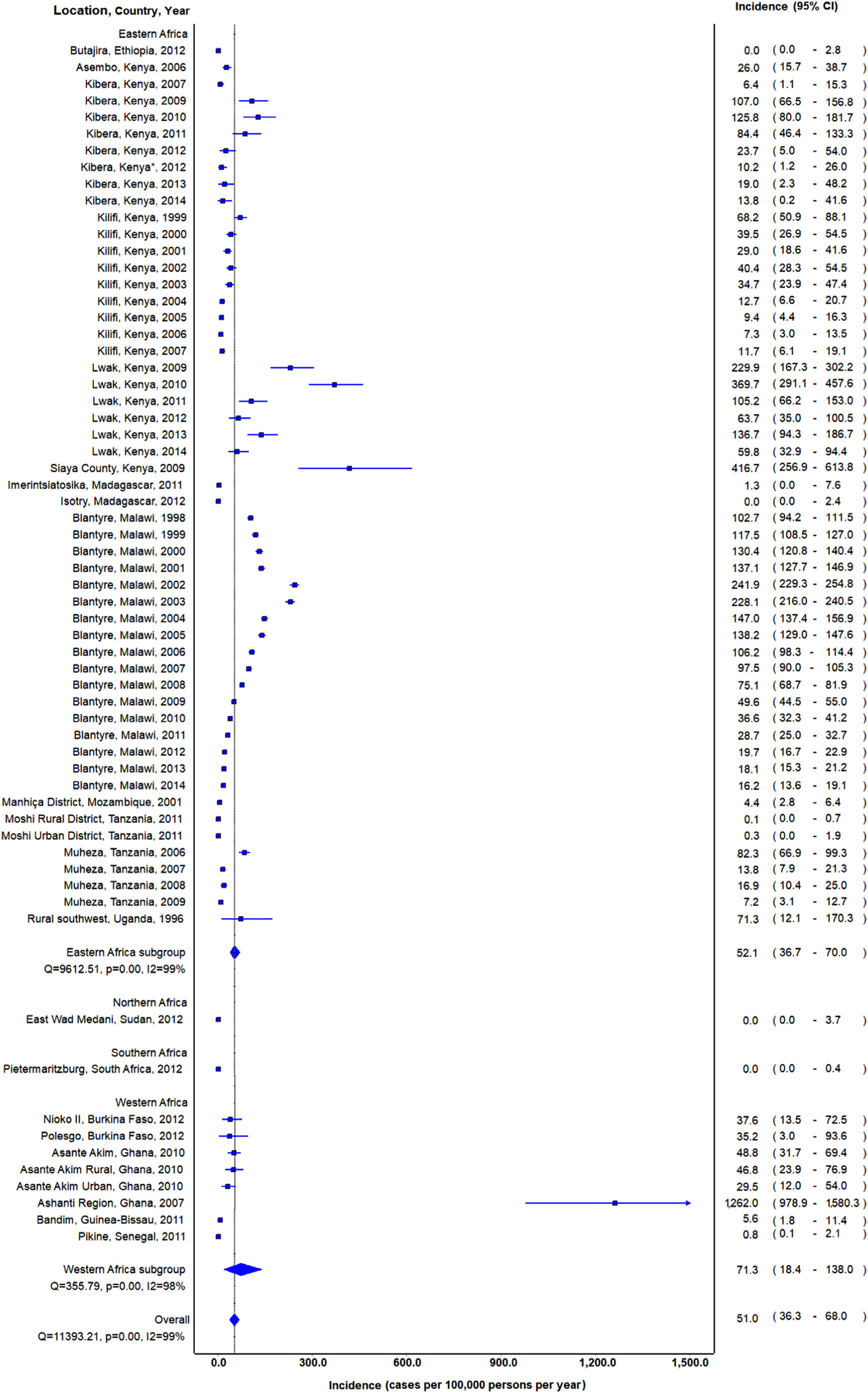
Forest plot of non-typhoidal *Salmonella* invasive disease incidence in Africa by United Nations sub-regions, 1996–2016 * Data from Marks et al. is same location and year as Verani et al.

Among 68 estimates of incidence, six (8.8%) reported no cases of iNTS isolated from a normally sterile site^25,27^ and the highest incidence reported was 1262.0 in Ghana (Supplement Table S1).^31^ Overall pooled incidence (95% CI) was 44.8 (31.5–60.5) per 100,000 persons per year. When stratified by region, pooled incidence was significantly higher in Africa than Asia, 51.0 (36.3–68.0) compared to 1.0 (0.2–2.5), respectively. Among sub-regions in Africa, pooled incidence was 71.3 (18.4–138.0) in Western Africa, 52.1 (36.7–70.0) in Eastern Africa, <0.1 (0.0–3.7) in Northern Africa, and <0.1 (0.0–0.4) in Southern Africa (Fig. 3). No included study reported estimates from Middle Africa. Among the three countries with the most estimates of incidence, pooled incidence was 85.6 (55.8–121.5), 56.7 (38.1–79.0), and 12.1 (0.0–32.4) in Malawi, Kenya, and Tanzania, respectively. Among the locations of Kibera, Kilifi, and Lwak, Kenya; and Blantyre, Malawi; where there were multiple consecutive years of incidence data, there was a pattern of lower incidence in more recent studies (Fig. 3).

By study design, pooled incidence among studies using hybrid surveillance was 42.2 (25.1–63.7), and among unadjusted sentinel surveillance studies was 45.5 (29.6–64.9). Eight studies provided age-stratified crude incidence estimates^23,26,27,30–32,34,35^ and five provided adjusted incidence using one or more multiplier (Table 2).^23,27,30,34,35^ Younger age groups between zero and five years consistently had higher iNTS incidence than older populations. The highest reported crude incidence among age-stratified studies was 4,133.2 among 1–11 month old infants in Siaya County, Kenya.^32^ Among the five studies using a multiplier, four (80.0%) used both facility coverage and enrollment capture adjustments^23,30,34,35^ and one (20.0%) facility coverage only.^27^ One study described adjusting the incidence to account for blood culture sensitivity but did not provide data for the adjusted rates.^24^

Three studies provided incidence by individual serovars in addition to iNTS incidence overall.^24,26,34^ Among these three studies, there were 20 estimates of incidence for *Salmonella* Typhimurium, 20 estimates of *Salmonella* Enteritidis (Supplement Figs. S1 and S2, respectively), and one estimate of *Salmonella* Heidelberg. The median (range) incidence of *Salmonella* Typhimurium was 68.8 (3.1–204.7) and 7.0 (0.8–55.7) for *Salmonella* Enteritidis. The single incidence estimate for *Salmonella* Heidelberg was 0.4 from Kenya in 2006.^34^

### *Prevalence of non-typhoidal* Salmonella *serovars*

Five (38.5%) of the 13 studies provided data on prevalence of iNTS serovars among isolates from normally sterile sites; all were studies located in Africa. Among the five studies, 8,726 (77.4%) of 11,271 iNTS were *Salmonella* Typhimurium, followed by 1919 (17.0%) *Salmonella* Enteritidis, and 10 (0.1%) *Salmonella* Dublin. The remaining 14 serovars each accounted for <0.1% of NTS reported (Table 3). For 588 (5.2%) isolates, serotyping was performed but serovars could not be determined, or the authors provided only the most common serovars and not all serovars that were identified.

## Discussion

Our systematic review of iNTS incidence demonstrated varying levels of incidence between countries, locations in close proximity, and consecutive years in the same location, displaying considerable heterogeneity in both place and time. Similar heterogeneity of incidence has also been observed for typhoid fever.^36^ Incidence in Africa was significantly higher than in Asia, and no data were available from other regions. Serovars isolated were predominately *Salmonella* Typhimurium and Enteritidis, accounting for more than 90% of all iNTS that were serotyped.

The pooled incidence estimate of 51 per 100,000 per year in Africa in our review was similar to one provided by a 2017 estimate^4^ but substantially lower to an estimate for 2010.^13^ Lower recent incidence estimates may reflect improvements in host risk factors for iNTS disease, including expanded coverage of HIV prevention and care services, and declining malaria incidence in Africa.^37,38^ It is possible that variations in the prevalence of host risk factors for iNTS disease such as HIV, malaria, and malnutrition, and presence or absence of key serovars and sequence types may contribute to the heterogeneity between and within each review. Additionally, the methods between reviews varied, with lower-quality national surveillance data used in previous reviews, as well as the application of differing extrapolation methods to estimate incidence in areas that lacked data.

Among studies stratifying iNTS incidence by age, children aged <5 years regularly had incidence rates higher than older children and adults, and incidence was highest among infants. Infants and younger children represent a key target for iNTS vaccines. However, data in eligible incidence studies lacked sufficient resolution to examine differences in incidence by narrower age bands during the first 12 months of life.

Since *Salmonella* Typhimurium and Enteritidis accounted for >90% of iNTS infections, a bivalent vaccine would address the majority of NTS serovars causing invasive disease. However, it is known that some NTS serovars demonstrate geographic localization and that given the small number of eligible studies, we cannot rule out the presence of unstudied locations where otherwise rare serovars predominate. Antimicrobial resistance in NTS causing invasive disease has recently been reviewed by others.^9^ The prevalence of antimicrobial resistance to widely used antimicrobial classes in NTS causing invasive disease further underscores the need for prevention interventions.

Our review had several limitations. First, there were substantial gaps in data available from the published literature. Robust incidence estimates data were not available for the majority of countries in Africa. Countries and areas with known high prevalence of HIV, malaria incidence, and malnutrition lacking data on iNTS are potential high priority targets for future studies. Hospital-based prevalence studies of NTS BSI could be used as a lower-cost alternative to more resource intensive population-based incidence studies to gain insights into the role of NTS as a cause of bacteremia in unstudied locations.^39^ Second, available data were subject to moderate or high risk of bias. Varying types and numbers of multipliers were used across studies. There is a need to establish a standard design for hybrid surveillance studies. We also observed substantial heterogeneity in our meta-analyses. Lastly, since high typhoidal and non-typhoidal *Salmonella* invasive disease incidence occur uncommonly at the same site,^5,6^ the inclusion of data from the Typhoid Fever Surveillance in Africa Program (TSAP) that targeted areas with known occurence of typhoid fever^27^ may have biased out review towards sites with less iNTS disease.

We found that iNTS incidence varies by region, location, age group, and over time. While a large number of *Salmonella enterica* serovars cause iNTS, *Salmonella* Typhimurium and Enteritidis predominate. Concerted efforts are needed to address the limited high-quality data available on iNTS disease incidence. Increased sentinel site surveillance, as well as prevalence studies, are needed to better understand iNTS epidemiology. Bivalent vaccines targeting *Salmonella* Typhimurium and Enteritidis have the potential to prevent considerable iNTS disease among African infants and children.

## Supplementary Material

Suppl Appendix C

Suppl Appendix D

Suppl Appendix B

Suppl Appendix A

## Figures and Tables

**Table 1:** Characteristics of included studies of non-typhoidal *Salmonella* incidence by United Nations sub-region, 1996 through 2016.

UNsub-region	Study location, Country	Study design	Years of data collection	Cultures	Inclusion age group	NTS serovar	Type of multiplier
Eastern Africa	Butajira, Ethiopia ^27^	Hybrid surveillance	2012–2014	Blood	Mixed ages	NTS	None
	Kilifi, Kenya ^30^	Hybrid surveillance	1996–2014	Blood	Mixed ages	NTS	F, E
	Kilifi, Kenya ^33^	Unadjusted sentinel site surveillance	1999–2007	Blood	Children	NTS	None
	Asembo, Kenya ^34^	Hybrid surveillance	2006–2009	Blood	Mixed ages	NTS *Salmonella* Enteritidis *Salmonella* Heidelberg *Salmonella* Typhimurium	F, E
	Kibera, Kenya ^34^	Hybrid surveillance	2007–2009	Blood	Mixed ages	NTS *Salmonella* Enteritidis *Salmonella* Typhimurium	F, E
	Lwak, Kenya ^35^	Hybrid surveillance	2009–2014	Blood	Mixed ages	NTS	F, E
	Kibera, Kenya ^35^	Hybrid surveillance	2009–2014	Blood	Mixed ages	NTS	F, E
	Siaya County, Kenya ^32^	Vaccine trial control arms	2009–2013	Blood	Children	NTS	None
	Kibera, Kenya ^27^	Hybrid surveillance	2012–2013	Blood	Mixed ages	NTS	F
	Imerintsiatosika, Madagascar ^27^	Hybrid surveillance	2011–2013	Blood	Mixed ages	NTS	F
	Isotry, Madagascar ^27^	Hybrid surveillance	2012–2013	Blood	Mixed ages	NTS	F
	Blantyre, Malawi ^24^	Unadjusted sentinel site surveillance	1998–2014	Blood;CSF	Mixed ages	NTS *Salmonella* Enteritidis *Salmonella* Typhimurium	T*
	Manhiça District, Mozambique ^26^	Unadjusted sentinel site surveillance	2001–2014	Blood;CSF	Children	NTS *Salmonella* Enteritidis *Salmonella* Typhimurium	None
	Muheza, Tanzania ^29^	Unadjusted sentinel site surveillance	2006–2010	Blood	Children	NTS	None
	Moshi Rural District, Tanzania ^27^	Hybrid surveillance	2011–2013	Blood	Mixed ages	NTS	F, E
	Moshi Urban District, Tanzania ^27^	Hybrid surveillance	2011–2013	Blood	Mixed ages	NTS	F, E
	Rural southwest, Uganda ^28^	Unadjusted sentinel site surveillance	1996–2007	Blood	Mixed ages	NTS	None
Northern Africa	East Wad Medani, Sudan ^27^	Hybrid surveillance	2012–2013	Blood	Mixed ages	NTS	F
Southern Africa	Pietermaritzburg, South Africa ^27^	Hybrid surveillance	2012–2014	Blood	Mixed ages	NTS	None
Western Africa	Nioko II, Burkina Faso ^27^	Hybrid surveillance	2012–2013	Blood	Mixed ages	NTS	F
	Polesgo, Burkina Faso ^27^	Hybrid surveillance	2012–2013	Blood	Mixed ages	NTS	F
	Ashanti Region, Ghana^31^	Hybrid surveillance	2007–2009	Blood	Children	NTS	None
	Asante Akim, Ghana ^23^	Hybrid surveillance	2010–2012	Blood	Children	NTS	F, E
	Asante Akim, Ghana^27^	Hybrid surveillance	2010–2012	Blood	Children	NTS	F
	Bandim, Guinea-Bissau ^27^	Hybrid surveillance	2011–2013	Blood	Mixed ages	NTS	F
	Pikine, Senegal ^27^	Hybrid surveillance	2011–2013	Blood	Mixed ages	NTS	None
Eastern Asia	Hechi, China ^25^	Unadjusted sentinel site surveillance	2001–2002	Blood	Mixed ages	NTS	None
South-eastern Asia	North Jakarta, Indonesia ^25^	Unadjusted sentinel site surveillance	2002–2003	Blood	Mixed ages	NTS	None
	Hue, Vietnam ^25^	Unadjusted sentinel site surveillance	2002–2003	Blood	Mixed ages	NTS	None
Southern Asia	Kolkata, India ^25^	Unadjusted sentinel site surveillance	2003–2004	Blood	Mixed ages	NTS	None
	Karachi, Pakistan ^25^	Unadjusted sentinel site surveillance	2002–2004	Blood	Children	NTS	None

NTS = Non-typhoidal *Salmonella;* CSF = Cerebrospinal fluid; *F* = Facility coverage; *E* = Enrollment capture; *T* = Test sensitivity

* Described adjusting incidence to account for blood culture sensitivity but did not provide data for the adjusted rates.

**Table 2:** Age stratified incidence and adjusted incidence of non-typhoidal Salmonella invasive disease by United Nations sub-region and year, 1996 through 2016.

UN sub-region	Study location, Country	Year surveillance started	Age stratified crude incidence, 100,000 per PYO	Type of multipliers	Age stratified adjusted or overall adjusted incidence, 100,000 per PYO
Eastern Africa	Kilifi, Kenya ^30^	1996	0–4y: 25.6; 5–14y: 1.9; > = 15y: 1.0	F, E	0–4y: 32.6; 5–14y: 2.4; > = 15y: 1.7
	Manhiça District, Mozambique ^26^	2001	0–11m: 217.7; 12–59m: 172.7; > = 60m: 7.8	NA	NR
	Asembo (rural), Kenya ^34^	2006	0–4y: 206.0; 5–9y: 53.0; 10–17y: 6.0; 18–49: 76.0; > 50: 58.0	F, E	0–4y: 2,085.0; 5–9y: 389.0; 10–17y: 24.0; 18–49y: 367.0; > 50y: 232.0; All ages: 580.0
	Kibera (urban), Kenya ^34^	2007	0–4y: 52.0; 5–9y: 12.0; 10–17y: 0.0; 18–49: 3.7; > 50: 0.0	F, E	0–4y: 260.0; 5–9y: 37.0; 10–17y: 0.0; 18–49y: 11.0; > 50y: 0.0; All ages: 57.0
	Siaya County, Kenya ^32^	2009	1–11m: 4,133.2; 12–23m: 2,253.5; 24–35m: 1,279.2; 36–70m: 733.8	NA	NR
	Lwak (rural), Kenya ^35^	2009	0–4y: 501.8 ^a^; 5–9y: 118.3 ^a^; 10–17y: 62.8 ^a^; 18–49: 115.7 ^a^; > 50: 69.2 ^a^	F, E	< 12m: 3,533.0 ^a^; 12–23m: 6,419.1 ^a^; 24–35m: 3,888.3 ^a^; 36–47m: 3,771.7 ^a^; 48–59m: 1,788.9 ^a^; 5–9y: 374.5 ^a^; 10–17y: 216.1 ^a^; 18–49y: 325.7 ^a^; > 50y: 249.5 ^a^ All ages: 1,428.7
	Kibera (urban), Kenya ^35^	2009	0–4y: 254.9 ^a^; 5–9y: 41.8 ^a^; 10–17y: 10.5 ^a^; 18–49: 28.0 ^a^; > 50: 0 ^a^	F, E	< 12m: 2,210.0 ^a^; 12–23m: 1,483.8 ^a^; 24–35m: 805.1 ^a^; 36–47m: 636.6 ^a^; 48–59m: 185.4 ^a^; 5–9y: 82.5 ^a^; 10–17y: 21.3 ^a^; 18–49y: 62.2 ^a^; > 50y: 0.0 ^a^ All ages: 185.5
	Lwak (rural), Kenya ^35^	2010	NR	F, E	1,927.3
	Kibera (urban), Kenya ^35^	2010	NR	F, E	218.5
	Lwak (rural), Kenya ^35^	2011	NR	F, E	608.5
	Kibera (urban), Kenya ^35^	2011	NR	F, E	220.5
	Imerintsiatosika, Madagascar ^27^	2011	0–1y: 77.7; 2–4y: 0.0; 5–14y: 0.0; > = 15y: 0.0	F	0–1y: 100.0; 2–4y: 0.0; 5–14y: 0.0; > = 15y: 0.0; All ages: 9.0
	Moshi Rural District, Tanzania ^27^	2011	0–1y: 0.0; 2–4y: 0.0; 5–14y: 0.0; > = 15y: 21.8	F, E	0–1y: 0.0; 2–4y: 0.0; 5–14y: 0.0; > = 15y: 28.0; All ages: 7.0
	Moshi Urban District, Tanzania ^27^	2011	0–1y: 336.1; 2–4y: 0.0; 5–14y: 0.0; > = 15y: 0.0	F, E	0–1y: 427.0; 2–4y: 0.0; 5–14y: 0.0; > = 15y: 0.0;
	Butajira, Ethiopia ^27^	2012	0–1y: 0.0; 2–4y: 0.0; 5–14y: 0.0; > = 15y: 0.0	NA	NR
	Kibera, Kenya ^27^	2012	0–1y: 49.2; 2–4y: 49.0; 5–14y: 17.5; > = 15y: 32.5	F	0–1y: 49.0; 2–4y: 49.0; 5–14y: 17.0; > = 15y: 33.0; All ages: 32.0
	Lwak (rural), Kenya ^35^	2012	NR	F, E	303.3
	Kibera (urban), Kenya ^35^	2012	NR	F, E	62.5
	Isotry, Madagascar ^27^	2012	0–1y: 0.0; 2–4y: 0.0; 5–14y: 0.0; > = 15y: 0.0	F	0–1y: 0.0; 2–4y: 0.0; 5–14y: 0.0; > = 15y: 0.0; All ages: 0.0
	Lwak (rural), Kenya ^35^	2013	NR	F, E	745.5
	Kibera (urban), Kenya ^35^	2013	NR	F, E	93.4
	Lwak (rural), Kenya ^35^	2014	NR	F, E	337.8
	Kibera (urban), Kenya ^35^	2014	NR	F, E	87.2
Northern Africa	East Wad Medani, Sudan ^27^	2012	0–1y: 0.0; 2–4y: 0.0; 5–14y: 0.0; > = 15y: 0.0	F	0–1y: 0.0; 2–4y: 0.0; 5–14y: 0.0; > = 15y: 0.0; All ages: 0.0
Southern Africa	Pietermaritzburg, South Africa ^27^	2012	0–1y: 0.0; 2–4y: 0.0; 5–14y: 0.0; > = 15y: 0.0	NA	NR
Western Africa	Ashanti Region, Ghana ^31^	2007	< 1m: 37.5; 1–11m: 862.6; 12–23m: 843.8; 24–35m: 337.5; 36–47m: 281.3; 48–60m: 56.3	NA	NR
	Asante Akim (urban), Ghana ^23^	2010	0–1y: 380.0; 2–4y: 316.4; 5–14y: 24.2	F, E	0–1y: 927.3; 2–4y: 769.9; 5–14y: 64.2; < 15y: 346.4
	Asante Akim (rural), Ghana ^23^	2010	0–1y: 966.2; 2–4y: 1,150.2; 5–14y: 46.6	F, E	0–1y: 2353.3; 2–4y: 2,808.4; 5–14y: 123.4; < 15y: 1,012.1
	Asante Akim, Ghana ^27^	2010	0–1y: 710.8; 2–4y: 782.3; 5–14y: 55.8	F	0–1y: 1,733.0; 2–4y: 1,908.0; 5–14y: 147.0; < 15y: 742.0
	Bandim, Guinea-Bissau ^27^	2011	0–1y: 96.2 2–4y: 25.9 5–14y: 18.0 > = 15y: 0.0	F	0–1y: 291.0; 2–4y: 53.0; 5–14y: 53.0; > = 15y: 0.0; All ages: 37.0
	Pikine, Senegal ^27^	2011	0–1y: 0.0 2–4y: 0.0 5–14y: 2.3 > = 15y: 3.6	NA	NR
	Nioko II, Burkina Faso ^27^	2012	0–1y: 143.1; 2–4y: 143.1; 5–14y: 61.4; > = 15y: 9.4	F	0–1y: 753.0; 2–4y: 753.0; 5–14y: 236.0; > = 15y: 35.0; All ages: 237.0
	Polesgo, Burkina Faso ^27^	2012	0–1y: 107.6; 2–4y: 201.6; 5–14y: 0.0 > = 15y: 20.3	F	0–1y: 431.0; 2–4y: 630.0; 5–14y: 0.0; > = 15y: 54.0; All ages: 144.0

Multipliers: F = Facility coverage: eligible participants not seeking care at facility; E = Enrollment: eligible participants did not have a blood culture collected; NA: Not applicable; NR: Not reported; m: month; y: year; PYO: person-years observed.

a Age stratified adjusted incidence data is for entire surveillance period from 2009 through 2014. Age stratified adjusted incidence was not reported for each individual year.

**Table 3:** Prevalence of non-typhoidal *Salmonella enterica* serovars in Africa, 1998 through 2016.

*Salmonella enterica* serovar	Cases	Proportion of isolates,%
Typhimurium	8,726	77.4
Enteritidis	1,919	17.0
Dublin	10	0.1
Heidelberg	5	< 0.1
Choleraesuis	4	< 0.1
Infantis	4	< 0.1
Virchow	3	< 0.1
Derby	2	< 0.1
Panama	2	< 0.1
Bovismorbificans	1	< 0.1
Hadar	1	< 0.1
Isangi	1	< 0.1
Kibusi	1	< 0.1
Senegal	1	< 0.1
Stanleyville	1	< 0.1
Umbilo	1	< 0.1
Urbana	1	< 0.1
Other *Salmonella* *	588	5.2
**Total**	**11,271**	**100.0**

* Serotyping performed but could not identify serovars or authors provided only most common serovars and did not describe all serovars that were serotyped.
