# Supplementary material for: Incidence of non-typhoidal Salmonella invasive disease: A systematic review and meta-analysis
Source: J Infect. Author manuscript; Available in PMC 2021 Nov 28. (PMC8627500; doi:10.1016/j.jinf.2021.06.029)
Supplement: Suppl Appendix B [file NIHMS1747329-supplement-Suppl_Appendix_B.pdf]

# iNTS incidence systematic review data abstraction

* Required

1. Name of reviewer *

Mark only one oval.

- ⬭ Christian Marchello
- ⬭ Elena Pettini
- ⬭ Fabio Fiorino
- ⬭ John Crump

2. First author's last name *

____

3. Publication year *

____

4. PubMed ID *

____

Study characteristics

5. Study country *

____

6. Study locality (city, district, or province) *

____

7. UN region *

https://unstats.un.org/unsd/methodology/m49/

Mark only one oval.

- Africa *Skip to question 14*
- Americas *Skip to question 15*
- Asia *Skip to question 16*
- Europe *Skip to question 17*
- Oceania *Skip to question 18*

Study characteristics

8. Study design *

Mark only one oval.

- Active surveillance, population-based without multipliers
- Active surveillance, population-based with multiplier
- Active surveillance, household-based without multipliers
- Active surveillance, household-based with multipliers
- Passive surveillance with catchment information (minimum incidence)
- Prospective observational
- Vaccine trial control arms
- Other: ______________________

9. Date data collection began *

If no specific day provided, use the first of the month; if only a year is provided, enter 1 January

Example: January 7, 2019

10. Date data collection ended *

If no specific day provided, use the last day of the month; if only a year is provided, enter 31 December

Example: January 7, 2019

11. Normally sterile site cultured *

Check all that apply.

- [ ] Blood
- [ ] Bone marrow
- [ ] CSF
- [ ] Abcess

Other: [ ] ____________________

12. Definition for culture request *

(e.g. fever >3 days)

____
____
____
____
____

13. Inclusion age group *

Based on inclusion criteria or age range data provided in results

Mark only one oval.

- ( ) Children only (<=15y)
- ( ) Adults only (>15y)
- ( ) Mixed ages

Skip to question 19

Africa sub-regions

14.
Mark only one oval.

- Eastern Africa
- Middle Africa
- Northern Africa
- Southern Africa
- Western Africa

Skip to question 8

Americas sub-regions

15.
Mark only one oval.

- Caribbean
- Central America
- Northern America
- South America

Skip to question 8

Asia sub-regions

16.
Mark only one oval.

- Central Asia
- Eastern Asia
- South-eastern Asia
- Southern Asia
- Western Asia

Skip to question 8

Europe sub-regions

17. *Mark only one oval.*

- ◯ Eastern Europe
- ◯ Northern Europe
- ◯ Southern Europe
- ◯ Western Europe

Skip to question 8

Oceania sub-regions

18. *Mark only one oval.*

- ◯ Australia and New Zealand
- ◯ Melanesia
- ◯ Micronesia
- ◯ Polynesia

Skip to question 8

Incidence data

19. Year that surveillance started *

____

20. Duration of surveillance (in months) *

____

21. Population under surveillance

Denominator of incidence calculation; 'Not provided' if only person-time is given

____

22. Person-time observed

N/A if not provided or applicable

____

23. Which NTS serovar is data available? *

Mark only one oval.

- ( ) NTS only (does not stratify by serovar) *Skip to question 24*
- ( ) Salmonella Typhimurium *Skip to question 25*
- ( ) Salmonella Enteritidis *Skip to question 26*
- ( ) Salmonella Heidelberg *Skip to question 27*
- ( ) Salmonella Dublin *Skip to question 28*
- ( ) Salmonella Newport *Skip to question 29*
- ( ) Salmonella Choleraesuis *Skip to question 30*
- ( ) Other *Skip to question 31*

Incidence data

24. Cases of NTS only (does not stratify by serovar)

Number cultured from normally sterile sites

____

Skip to question 32

Incidence data

25. Cases of Salmonella Typhimurium

Number cultured from normally sterile sites

____

Skip to question 32

Incidence data

26. Cases of Salmonella Enteritidis

Number cultured from normally sterile sites

Skip to question 32

Incidence data

27. Cases of Salmonella Heidelberg

Number cultured from normally sterile sites

Skip to question 32

Incidence data

28. Cases of Salmonella Dublin

Number cultured from normally sterile sites

Skip to question 32

Incidence data

29. Cases of Salmonella Newport

Number cultured from normally sterile sites

Skip to question 32

Incidence data

30. Cases of Salmonella Choleraesuis

Number cultured from normally sterile sites

Skip to question 32

Incidence data

31. Cases of other non-typhoidal Salmonella

Specify serovar and number cultured from normally sterile sites

____

Skip to question 32

Age-stratified incidence data

32. Is age stratified incidence provided?

Mark only one oval.

- ( ) Yes *Skip to question 33*
- ( ) No *Skip to question 34*

Skip to question 34

Age-stratified incidence data

33. Age stratified incidence

Age group and incidence

____
____
____
____
____

Adjusted incidence data

34. Is adjusted incidence provided?

Mark only one oval.

- ( ) Yes *Skip to question 35*
- ( ) No *Skip to question 37*

Skip to question 37

Adjusted incidence data

35. Type of multipliers

Check all that apply.

- [ ] Healthcare utilization: eligible participants not seeking care at facility
- [ ] Enrollment: eligible participants did not have a blood culture collected
- [ ] Test sensitivity: adjustment for culture
- [ ] Seasonality
- [ ] None provided

Other: [ ] ____________________

36. Adjusted incidence

incidence (cases/population/time; 10/100,000/year)

Skip to question 37

Scan of reference list

37. List of references needing check

Based on context in which citation was used and the title of article

Notes

38. Notes on the article, if necessary

This content is neither created nor endorsed by Google.

Google Forms
