# Supplementary material for: Incidence of non-typhoidal Salmonella invasive disease: A systematic review and meta-analysis
Source: J Infect. Author manuscript; Available in PMC 2021 Nov 28. (PMC8627500; doi:10.1016/j.jinf.2021.06.029)
Supplement: Suppl Appendix A [file NIHMS1747329-supplement-Suppl_Appendix_A.pdf]

Supplementary Appendix A – search strategy

PubMed 08 May 2020

((((((nontyph* OR non-typh* OR "iNTS" OR "salmonella group" OR "salmonellosis" OR typhimurium OR enteritidis OR (salmonella AND Heidelberg) OR (salmonella AND Dublin) OR (salmonella AND Choleraesuis) OR (salmonella AND Newport) OR (salmonella AND Virchow) OR (salmonella AND Concord) OR (salmonella AND Brancaster) OR (salmonella AND freetown) OR (salmonella AND "Infantis") OR (salmonella AND Isangi))))) AND ((incidence OR epidemiolog* OR burden))) NOT (("Animals, Wild"[MeSH Terms] OR cell OR mouse OR rat OR rodent* OR bat OR bats OR cattle)) AND hasabstract[text] AND Humans[Mesh])

Search Details

Query Translation:

```
(((nontyphi[All Fields] OR nontyphodial[All Fields] OR nontyphoid[All Fields] OR nontyphoidal[All Fields] OR nontyphoidalserovars[All Fields]
OR nontyphoideal[All Fields] OR nontyphoidic[All Fields] OR nontyphosa[All Fields] OR nontyphosal[All Fields] OR nontyphus[All Fields]) OR
[illegible]
```

Search URL

Result:

3202

Translations:

salmonella	"salmonella"[MeSH Terms] OR "salmonella"[All Fields]
Newport	"Newport"[Journal] OR "newport"[All Fields]
Concord	"Afr Concord"[Journal] OR "concord"[All Fields]
incidence	"epidemiology"[Subheading] OR "epidemiology"[All Fields] OR "incidence"[All Fields] OR "incidence"[MeSH Terms]
cell	"cells"[MeSH Terms] OR "cells"[All Fields] OR "cell"[All Fields]
mouse	"mice"[MeSH Terms] OR "mice"[All Fields] OR "mouse"[All Fields]
rat	"rats"[MeSH Terms] OR "rats"[All Fields] OR "rat"[All Fields]
bat	"Behav Anal Today"[Journal] OR "bat"[All Fields]
bats	"chiroptera"[MeSH Terms] OR "chiroptera"[All Fields] OR "bats"[All Fields]
cattle	"cattle"[MeSH Terms] OR "cattle"[All Fields]
Humans[Mesh]	"humans"[MeSH Terms]

Database:

PubMed

User query:

((((((nontyph* OR non-typh* OR "iNTS" OR "salmonella group" OR "salmonellosis" OR typhimurium OR enteritidis OR (salmonella AND Heidelberg) OR (salmonella AND Dublin) OR (salmonella AND Choleraesuis) OR (salmonella AND Newport) OR (salmonella AND Virchow) OR (salmonella AND Concord) OR (salmonella AND Brancaster) OR (salmonella AND freetown) OR (salmonella AND "Infantis") OR (salmonella AND Isangi))))) AND ((incidence OR epidemiolog* OR burden))) NOT (("Animals, Wild"[MeSH Terms] OR cell OR mouse OR rat OR rodent* OR bat OR bats OR cattle)) AND hasabstract[text] AND Humans[Mesh])

Web of Science 08 May 2020

(TS=(nontyph* OR non-typh* OR "iNTS" OR "salmonella group" OR "salmonellosis" OR typhimurium OR enteritidis) OR TS=(salmonella AND Heidelberg) OR TS=(salmonella AND Dublin) OR TS=(salmonella AND Choleraesuis) OR TS=(salmonella AND Newport) OR TS=(salmonella AND Virchow) OR TS=(salmonella AND Concord) OR TS=(salmonella AND Brancaster) OR TS=(salmonella AND "freetown") OR TS=(salmonella AND "Infantis") OR TS=(salmonella AND Isangi)) AND (TS=(incidence OR epidemiolog* OR burden)) NOT (TS=(cell OR mouse OR rat OR rodent* OR bat OR bats OR cattle))
Refined by: [excluding] DOCUMENT TYPES: (ABSTRACT OR MEETING OR LETTER OR BOOK OR EDITORIAL OR NEWS OR BIOGRAPHY OR REPORT OR REFERENCE MATERIAL) AND [excluding] RESEARCH AREAS: (GENETICS HEREDITY OR MATHEMATICS OR PLANT SCIENCES OR BUSINESS ECONOMICS OR EVOLUTIONARY BIOLOGY OR ANATOMY MORPHOLOGY OR BIODIVERSITY CONSERVATION OR SOCIAL ISSUES OR NEUROSCIENCES NEUROLOGY OR MATHEMATICAL COMPUTATIONAL BIOLOGY OR MEDICAL LABORATORY TECHNOLOGY OR ENDOCRINOLOGY METABOLISM OR BEHAVIORAL SCIENCES OR CARDIOVASCULAR SYSTEM CARDIOLOGY OR SOCIOLOGY OR COMPUTER SCIENCE OR MARINE FRESHWATER BIOLOGY) AND [excluding] RESEARCH AREAS: (VETERINARY SCIENCES OR AGRICULTURE OR GOVERNMENT LAW OR METEOROLOGY ATMOSPHERIC SCIENCES OR INSTRUMENTS INSTRUMENTATION OR UROLOGY NEPHROLOGY OR ONCOLOGY OR DERMATOLOGY OR ENGINEERING)
Databases= WOS, BCI, CCC, INSPEC, MEDLINE, SCIELO Timespan=All years Search language=Auto

Web of Science

Clarivate Analytics

Search History

All Databases

Set	Results	Save History / Create Alert / Open Saved History	Combine Sets (AND / OR, Combine)	Delete Sets (Select All, Delete)
# 4	2,590	(TS=(nontyph* OR non-typh* OR "iNTS" OR "salmonella group" OR "salmonellosis" OR typhimurium OR enteritidis) OR TS=(salmonella AND Heidelberg) OR TS=(salmonella AND Dublin) OR TS=(salmonella AND Choleraesuis) OR TS=(salmonella AND Newport) OR TS=(salmonella AND Virchow) OR TS=(salmonella AND Concord) OR TS=(salmonella AND Brancaster) OR TS=(salmonella AND "freetown") OR TS=(salmonella AND "Infantis") OR TS=(salmonella AND Isangi)) AND (TS=(incidence OR epidemiolog* OR burden)) NOT (TS=(cell OR mouse OR rat OR rodent* OR bat OR bats OR cattle))<br>**Refined by:** [excluding] **DOCUMENT TYPES:** (ABSTRACT OR MEETING OR LETTER OR BOOK OR EDITORIAL OR NEWS OR BIOGRAPHY OR REPORT OR REFERENCE MATERIAL) AND [excluding] **RESEARCH AREAS:** (GENETICS HEREDITY OR MATHEMATICS OR PLANT SCIENCES OR BUSINESS ECONOMICS OR EVOLUTIONARY BIOLOGY OR ANATOMY MORPHOLOGY OR BIODIVERSITY CONSERVATION OR SOCIAL ISSUES OR NEUROSCIENCES NEUROLOGY OR MATHEMATICAL COMPUTATIONAL BIOLOGY OR MEDICAL LABORATORY TECHNOLOGY OR ENDOCRINOLOGY METABOLISM OR BEHAVIORAL SCIENCES OR CARDIOVASCULAR SYSTEM CARDIOLOGY OR SOCIOLOGY OR COMPUTER SCIENCE OR MARINE FRESHWATER BIOLOGY) AND [excluding] **RESEARCH AREAS:** (VETERINARY SCIENCES OR AGRICULTURE OR GOVERNMENT LAW OR METEOROLOGY ATMOSPHERIC SCIENCES OR INSTRUMENTS INSTRUMENTATION OR UROLOGY NEPHROLOGY OR ONCOLOGY OR DERMATOLOGY OR ENGINEERING)<br>*Databases= WOS, BCI, CCC, INSPEC, MEDLINE, SCIELO Timespan=All years*<br>*Search language=Auto*	☐	☐
# 3	4,709	(TS=(nontyph* OR non-typh* OR "iNTS" OR "salmonella group" OR "salmonellosis" OR typhimurium OR enteritidis) OR TS=(salmonella AND Heidelberg) OR TS=(salmonella AND Dublin) OR TS=(salmonella AND Choleraesuis) OR TS=(salmonella AND Newport) OR TS=(salmonella AND Virchow) OR TS=(salmonella AND Concord) OR TS=(salmonella AND Brancaster) OR TS=(salmonella AND "freetown") OR TS=(salmonella AND "Infantis") OR TS=(salmonella AND Isangi)) AND (TS=(incidence OR epidemiolog* OR burden)) NOT (TS=(cell OR mouse OR rat OR rodent* OR bat OR bats OR cattle))<br>**Refined by:** [excluding] **DOCUMENT TYPES:** (ABSTRACT OR MEETING OR LETTER OR BOOK OR EDITORIAL OR NEWS OR BIOGRAPHY OR REPORT OR REFERENCE MATERIAL) AND [excluding] **RESEARCH AREAS:** (GENETICS HEREDITY OR MATHEMATICS OR PLANT SCIENCES OR BUSINESS ECONOMICS OR EVOLUTIONARY BIOLOGY OR ANATOMY MORPHOLOGY OR BIODIVERSITY CONSERVATION OR SOCIAL ISSUES OR NEUROSCIENCES NEUROLOGY OR MATHEMATICAL COMPUTATIONAL BIOLOGY OR MEDICAL LABORATORY TECHNOLOGY OR ENDOCRINOLOGY METABOLISM OR BEHAVIORAL SCIENCES OR CARDIOVASCULAR SYSTEM CARDIOLOGY OR SOCIOLOGY OR COMPUTER SCIENCE OR MARINE FRESHWATER BIOLOGY)<br>*Databases= WOS, BCI, CCC, INSPEC, MEDLINE, SCIELO Timespan=All years*<br>*Search language=Auto*	☐	☐
# 2	8,903	(TS=(nontyph* OR non-typh* OR "iNTS" OR "salmonella group" OR "salmonellosis" OR typhimurium OR enteritidis) OR TS=(salmonella AND Heidelberg) OR TS=(salmonella AND Dublin) OR TS=(salmonella AND Choleraesuis) OR TS=(salmonella AND Newport) OR TS=(salmonella AND Virchow) OR TS=(salmonella AND Concord) OR TS=(salmonella AND Brancaster) OR TS=(salmonella AND "freetown") OR TS=(salmonella AND "Infantis") OR TS=(salmonella AND Isangi)) AND (TS=(incidence OR epidemiolog* OR burden)) NOT (TS=(cell OR mouse OR rat OR rodent* OR bat OR bats OR cattle))<br>**Refined by:** [excluding] **DOCUMENT TYPES:** (ABSTRACT OR MEETING OR LETTER OR BOOK OR EDITORIAL OR NEWS OR BIOGRAPHY OR REPORT OR REFERENCE MATERIAL)<br>*Databases= WOS, BCI, CCC, INSPEC, MEDLINE, SCIELO Timespan=All years*<br>*Search language=Auto*	☐	☐
# 1	10,807	(TS=(nontyph* OR non-typh* OR "iNTS" OR "salmonella group" OR "salmonellosis" OR typhimurium OR enteritidis) OR TS=(salmonella AND Heidelberg) OR TS=(salmonella AND Dublin) OR TS=(salmonella AND Choleraesuis) OR TS=(salmonella AND Newport) OR TS=(salmonella AND Virchow) OR TS=(salmonella AND Concord) OR TS=(salmonella AND Brancaster) OR TS=(salmonella AND "freetown") OR TS=(salmonella AND "Infantis") OR TS=(salmonella AND Isangi)) AND (TS=(incidence OR epidemiolog* OR burden)) NOT (TS=(cell OR mouse OR rat OR rodent* OR bat OR bats OR cattle))<br>*Databases= WOS, BCI, CCC, INSPEC, MEDLINE, SCIELO Timespan=All years*<br>*Search language=Auto*	☐	☐
			AND / OR<br>Combine	Select All<br>✕ Delete

Clarivate
Accelerating innovation

© 2020 Clarivate Copyright notice Terms of use Privacy statement Cookie policy

Sign up for the Web of Science newsletter Follow us

Embase Classic+Embase (1947 to 2020 May 08) + Ovid MEDLINE and Epub Ahead of Print, In-Process & Other Non-Indexed Citations, Daily and Versions (1946 to May 08, 2020)

Search History (42) View Saved

# ▲	Searches	Results	Type	Actions	Annotations
1	(salmonella and typhimurium).af.	81334	Advanced	Display Results More	
2	(salmonella and enteritidis).af.	17777	Advanced	Display Results More	
3	(salmonella and Heidelberg).af.	2564	Advanced	Display Results More	
4	(salmonella and Dublin).af.	2982	Advanced	Display Results More	
5	(salmonella and Choleraesuis).af.	1890	Advanced	Display Results More	
6	(salmonella and newport).af.	1582	Advanced	Display Results More	
7	(salmonella and virchow).af.	882	Advanced	Display Results More	
8	(salmonella and concord).af.	77	Advanced	Display Results More	
9	(salmonella and Brancaster).af.	35	Advanced	Display Results More	
10	(salmonella and freetown).af.	15	Advanced	Display Results More	
11	(salmonella and Infantis).af.	1761	Advanced	Display Results More	
12	(salmonella and Isangi).af.	73	Advanced	Display Results More	
13	remove duplicates from 3	1962	Advanced	Display Results More	
14	remove duplicates from 4	1842	Advanced	Display Results More	
15	remove duplicates from 5	1185	Advanced	Display Results More	
16	remove duplicates from 6	1005	Advanced	Display Results More	
17	remove duplicates from 7	547	Advanced	Display Results More	
18	remove duplicates from 8	51	Advanced	Display Results More	
19	remove duplicates from 9	21	Advanced	Display Results More	
20	remove duplicates from 10	11	Advanced	Display Results More	
21	remove duplicates from 11	1132	Advanced	Display Results More	
22	remove duplicates from 12	43	Advanced	Display Results More	
23	1 or 2 or 13 or 14 or 15 or 16 or 17 or 18 or 19 or 20 or 21 or 22	97944	Advanced	Display Results More	
24	nontyph*.af.	1834	Advanced	Display Results More	
25	non-typh*.af.	2873	Advanced	Display Results More	
26	salmonellosis.af.	30614	Advanced	Display Results More	
27	"iNTS".af.	2188	Advanced	Display Results More	
28	(salmonella adj7 group).af.	2750	Advanced	Display Results More	
29	remove duplicates from 24	1104	Advanced	Display Results More	
30	remove duplicates from 25	1778	Advanced	Display Results More	
31	remove duplicates from 27	1598	Advanced	Display Results More	
32	remove duplicates from 28	1824	Advanced	Display Results More	
33	23 or 26 or 29 or 30 or 31 or 32	119814	Advanced	Display Results More	
34	limit 33 to human	36354	Advanced	Display Results More	
35	limit 34 to journal [Limit not valid in Ovid MEDLINE(R),Ovid MEDLINE(R) Daily Update,Ovid MEDLINE(R) In-Process,Ovid MEDLINE(R) Publisher; records were retained]	36217	Advanced	Display Results More	
36	(incidence or epidemiolog* or burden).af.	6055510	Advanced	Display Results More	
37	limit 36 to human	5090096	Advanced	Display Results More	
38	limit 37 to journal [Limit not valid in Ovid MEDLINE(R),Ovid MEDLINE(R) Daily Update,Ovid MEDLINE(R) In-Process,Ovid MEDLINE(R) Publisher; records were retained]	5080938	Advanced	Display Results More	
39	(35 and 38) not (cell or mouse or rat or rodent* or bat or bats or cattle or papillomavirus or "HPV" or aquatic or marine or wild* or genome or "pet" or "pets" or cancer or diabetes).af.	8266	Advanced	Display Results More	
40	limit 39 to (article or clinical study or clinical trial, all or clinical trial, phase i or clinical trial, phase ii or clinical trial, phase iii or clinical trial, phase iv or journal article or observational study or randomized controlled trial) [Limit not valid in Embase,Ovid MEDLINE(R),Ovid MEDLINE(R) Daily Update,Ovid MEDLINE(R) In-Process,Ovid MEDLINE(R) Publisher; records were retained]	7113	Advanced	Display Results More	
41	limit 40 to abstracts	5324	Advanced	Display Results More	
42	remove duplicates from 41	3987	Advanced	Display Results More	

Contract

Save Remove Combine with: AND OR Deduplicate
